# Restricted Arm Swing Affects Gait Stability and Increased Walking Speed Alters Trunk Movements in Children with Cerebral Palsy

**DOI:** 10.3389/fnhum.2016.00354

**Published:** 2016-07-15

**Authors:** Tijs Delabastita, Kaat Desloovere, Pieter Meyns

**Affiliations:** ^1^Department of Rehabilitation Sciences, Faculty of Kinesiology and Rehabilitation Sciences, Katholieke Universiteit (KU) LeuvenHeverlee, Belgium; ^2^Clinical Motion Analysis Laboratory, CERM, University Hospital LeuvenLeuven, Belgium; ^3^Department of Rehabilitation Medicine, MOVE Research Institute Amsterdam, Vrije Universiteit, University Medical CenterAmsterdam, Netherlands

**Keywords:** cerebral palsy, gait, stability, walking speed, trunk movements, arm swing

## Abstract

Observational research suggests that in children with cerebral palsy, the altered arm swing is linked to instability during walking. Therefore, the current study investigates whether children with cerebral palsy use their arms more than typically developing children, to enhance gait stability. Evidence also suggests an influence of walking speed on gait stability. Moreover, previous research highlighted a link between walking speed and arm swing. Hence, the experiment aimed to explore differences between typically developing children and children with cerebral palsy taking into account the combined influence of restricting arm swing and increasing walking speed on gait stability. Spatiotemporal gait characteristics, trunk movement parameters and margins of stability were obtained using three dimensional gait analysis to assess gait stability of 26 children with cerebral palsy and 24 typically developing children. Four walking conditions were evaluated: (i) free arm swing and preferred walking speed; (ii) restricted arm swing and preferred walking speed; (iii) free arm swing and high walking speed; and (iv) restricted arm swing and high walking speed. Double support time and trunk acceleration variability increased more when arm swing was restricted in children with bilateral cerebral palsy compared to typically developing children and children with unilateral cerebral palsy. Trunk sway velocity increased more when walking speed was increased in children with unilateral cerebral palsy compared to children with bilateral cerebral palsy and typically developing children and in children with bilateral cerebral palsy compared to typically developing children. Trunk sway velocity increased more when both arm swing was restricted and walking speed was increased in children with bilateral cerebral palsy compared to typically developing children. It is proposed that facilitating arm swing during gait rehabilitation can improve gait stability and decrease trunk movements in children with cerebral palsy. The current results thereby partly support the suggestion that facilitating arm swing in specific situations possibly enhances safety and reduces the risk of falling in children with cerebral palsy.

## Introduction

The forelimbs have a clear locomotor function in quadrupedal walking. In human walking, this function most likely changed as the upper limbs do not make contact to the ground during upright walking. Irrespective of its quadrupedal neural base (Jackson, 1983; Dietz and Michel, 2009; Dominici et al., 2011), research indicates that arm swing facilitates balance recovery following a perturbation (Bruijn et al., 2010; Pijnappels et al., 2010). Moreover, the typical anti-phase arm swing pattern is suggested to reduce the energetic cost of human walking (Collins et al., 2009; Yizhar et al., 2009; Meyns et al., 2014).

In pathological populations, the arm swing pattern can be affected or altered during gait, which could result in changes in the function of the arm swing. Altered arm swing patterns have been reported in children with cerebral palsy. Cerebral palsy is a group of permanent disorders of the development of movement and posture, causing activity limitation, that are attributed to non-progressive disturbances that occur in the developing fetal or infant brain (Rosenbaum et al., 2007). Previous research found that arm swing amplitude was decreased on the hemiplegic side compared to the non-hemiplegic side in children with unilateral cerebral palsy (Meyns et al., 2011). Furthermore, children with bilateral cerebral palsy showed increased shoulder abduction and both children with unilateral as well as bilateral cerebral palsy walked with more elbow flexion compared to typically developing children (Romkes et al., 2007; Galli et al., 2014; Meyns et al., 2014). Moreover, the altered arm swing amplitude and arm posture changed inter-limb coordination in children with cerebral palsy (Meyns et al., 2012b). Children with cerebral palsy showed less stable coordination patterns and altered arm-leg movement frequency ratios compared to typically developing children (Meyns et al., 2012b).

While several changes of arm swing patterns have been reported in children with cerebral palsy, experimental evidence investigating the cause for these findings is still lacking. Nevertheless, such evidence should facilitate a more targeted therapeutic approach. For instance, previous correlational research suggested that altered arm swing in children with cerebral palsy plays an increased role in maintaining gait stability compared to typically developing children (Meyns et al., 2012a). As such, facilitating arm swing during gait rehabilitation could enhance safety, reduce the risk of falling and complement balance training in children with cerebral palsy. Therefore, the current experimental study aimed to examine the influence of restricting arm swing on gait stability in typically developing children, children with bilateral cerebral palsy and children with unilateral cerebral palsy. It is hypothesized that gait stability would decrease more in children with cerebral palsy compared to typically developing children when arm swing is restricted. Moreover, children with bilateral cerebral palsy are expected to present more gait instability because of bilateral involvement. Indeed, research previously suggested that children with bilateral cerebral palsy have more problems to generate situation-specific neuromuscular responses to maintain postural stability compared to children with unilateral cerebral palsy (Woollacott and Shumway-Cook, 2005). Therefore, it is hypothesized that gait stability would decrease more in children with bilateral cerebral palsy compared to children with unilateral cerebral palsy when arm swing is restricted.

Additionally, other authors previously suggested a possible influence of walking speed on gait stability. However, the exact relationship remains unclear (Dingwell and Marin, 2006; England and Granata, 2007; Bruijn et al., 2009, 2013b), especially in children with cerebral palsy. Therefore, current study aimed to examine the influence of increasing walking speed on gait stability in typically developing children, children with bilateral cerebral palsy and children with unilateral cerebral palsy. It is hypothesized that gait stability would decrease more in children with cerebral palsy compared to typically developing children when walking speed is increased. Furthermore, it is hypothesized that gait stability would decrease more in children with bilateral cerebral palsy compared to children with unilateral cerebral palsy when walking speed is increased.

Finally, a strong reciprocal influence between arm swing and walking speed was previously reported in children with cerebral palsy (Meyns et al., 2011). Therefore, the current study aimed to examine the influence of restricting arm swing combined with increasing walking speed influences on gait stability in typically developing children, children with bilateral cerebral palsy and children with unilateral cerebral palsy. It is expected that gait stability would decrease more in children with cerebral palsy compared to typically developing children when both arm swing is restricted and walking speed is increased. Moreover, it is hypothesized that gait stability would decrease more in children with bilateral cerebral palsy compared to children with unilateral cerebral palsy when both arm swing is restricted and walking speed is increased. Additionally, it is hypothesized that the influence of restricting arm swing combined with increasing walking speed is larger compared to the isolated influence of these tasks.

## Materials and methods

### Participants

Twenty-six children with cerebral palsy (age range 4–12 years) and 24 typically developing children (age range 5–12 years) were included in the study. The cerebral palsy group consisted of 11 children with unilateral cerebral palsy and 15 children with bilateral cerebral palsy, recruited from the Clinical Motion Analysis Laboratory of the U.Z. Leuven (Pellenberg). The children with cerebral palsy were only included in the study if they were diagnosed with the predominantly spastic type of cerebral palsy. Diagnosis and type of cerebral palsy were determined by a multidisciplinary team of neuropediatricians, pediatric orthopedicians, and rehabilitation physicians after neurological examination (including magnetic resonance imaging). The participants had to be able to walk without assistive devices and were only allowed to participate if they showed enough cooperation to follow the instructions concerning the walking trials. The children were excluded if they underwent Botulinum Toxin A treatment within the past 6 months or if they previously underwent lower limb surgery. The local ethical committee (Commissie Medische Ethiek KU Leuven) approved all experiments (approval number S51498). In accordance with the Declaration of Helsinki, written informed consent was obtained of the participants' parents.

### Protocol

Three-dimensional total-body kinematic data (100 Hz) were captured by an eight camera Vicon system (Oxford Metrics, Oxford, UK) to detect the reflective markers placed on the participant's skin. Similarly to Romkes et al. (2007), 34 reflective markers were used (Romkes et al., 2007). All children were first asked to walk at a self-selected speed along the 10 m walkway on a straight line with no restricted arm swing (“free arm swing and preferred walking speed”) or with the arms crossed in front of the body to restrict arm swing (“restricted arm swing and preferred walking speed”). Subsequently, the children were asked to walk as fast as possible with normal arm swing (“free arm swing and high walking speed”) and restricted arm swing (“restricted arm swing and high walking speed”). Three successful trials were recorded in each condition. A trial was considered successful when at least four consecutive foot strikes with full-marker-visibility were recorded. A trial was not retained if the participant made excessive movements of the head, arms or trunk unrelated to walking. Before recording the data, each participant completed some practice trials.

### Data processing

The marker coordinates were filtered and smoothed using Woltring's quintic spline routine with a predicted mean-squared error of 15. Further processing in Workstation (Vicon Workstation 5.2 beta 20, Oxford Metrics, Oxford, UK) and Polygon (version 3.1, Oxford Metrics, Oxford, UK) consisted of defining gait cycles and calculating spatiotemporal gait parameters. In children with cerebral palsy, the most affected side was defined as the side on which the highest median spasticity score (Modified Ashworth Scale) was obtained in the lower limb. In typically developing children, the least affected side was defined as their dominant side.

Various outcome measures were assessed to determine the children's stability during walking. In accordance with recent literature concerning stability measures in experimental situations, several spatiotemporal parameters were calculated (Bruijn et al., 2013a). Double support time was defined as the time of the gait cycle where both feet were in contact with the ground. Step length was calculated as the distance along the line of progression from the opposite foot contact to the current foot contact. Equivalently, step width was described as the distance normal to the line of progression, from the toe marker on one foot to the toe marker on the opposite foot when initial foot contact occurs. Last, stride length is the distance along the line of progression from current foot contact to the next foot contact. Double support time was normalized to total stride time. Step width, as well as step length and stride length, were normalized to the participant's height. Only values of the most affected side were retained for further analysis.

Furthermore, maximal amplitude, maximum velocity and maximum acceleration values for trunk sway and trunk rotation were calculated for all trials. The maximal amplitude was defined as the angle between the maximal value of trunk excursion on the most affected side and the maximal value of trunk excursion on the least affected side in one trial. Trunk sway was quantified in the frontal plane by calculating the angle between the axis through the C7 marker and the sacrum marker and the vertical axis. Similarly, the angle between the axis through the shoulder markers and the axis through the pelvic markers in the coronal plane was determined to evaluate trunk rotations.

**Figure 1:**
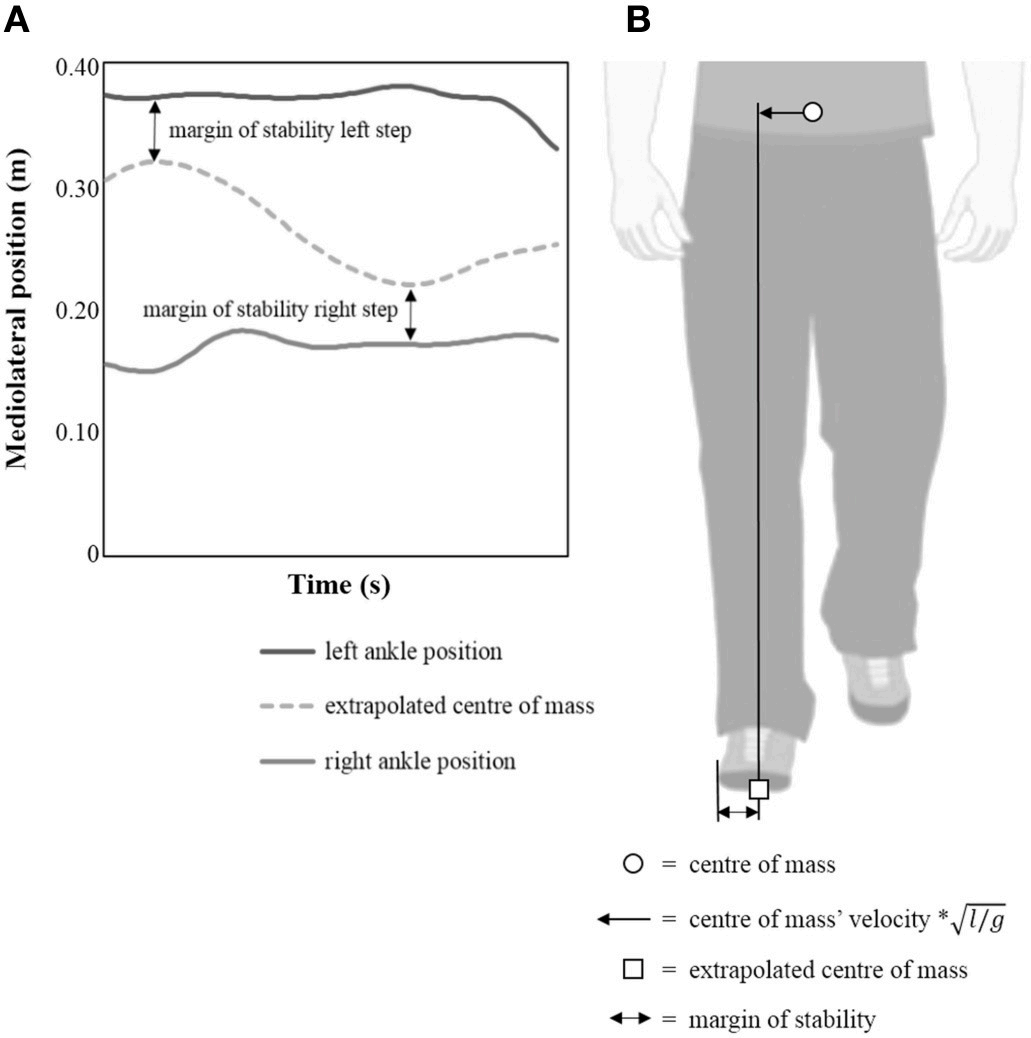
**Comprehensive illustration of the margin of stability as a measure of dynamic gait stability. (A)** schematic representation of the “extrapolated center of mass”-position and the position of the lateral ankle markers. **(B)** illustration of center of mass' position, “extrapolated center of mass”-vector and margin of stability-calculations.

In addition to the spatiotemporal parameters and the kinematic trunk data, the margin of stability was used as a measure of gait stability. The margin of stability is specifically developed as a measure of dynamic stability (Pai and Patton, 1997; Hof et al., 2005). Recently, this measure has often been used to predict gait stability in different subject groups (Denommé et al., 2014; Lin et al., 2015; Matsubara et al., 2015; Nagano et al., 2015; Simon et al., 2015) including children with cerebral palsy (Dixon et al., 2016). It is calculated as the shortest distance from the extrapolated center of mass to the borders of the base of support (Pai and Patton, 1997; Hof et al., 2005; Hof, 2008). With regard to the inverted pendulum model, a condition is considered to be stable when the vertical projection of the body center of mass is kept within the boundaries of the base of support in general static situations. However, the extrapolated center of mass encompasses both the current position and velocity of the center of mass. Therefore, the margin of stability extends the inverted pendulum model of stability in static situations to dynamic situations. It provides a model of gait stability with a strong biomechanical base (Bruijn et al., 2013a). Following the approach described by Hak et al. (2013), the extrapolated center of mass was calculated by adding the velocity and a factor √(l/g) to the position of the subject's center of mass. The center of mass was computed from the position and the relative weight from the segments of the PlugInGait-model. This method was found to be reliable (Gard et al., 2004). The parameter l represents the maximal height of the subject's calculated center of mass and g represents the gravitational acceleration. A schematic illustration of the margin of stability as a measure of gait stability is depicted in Figure 1. The margin of stability was assessed in the frontal plane by subtracting the maximum absolute extrapolated center of mass-value from the current lateral ankle position during foot contact (Hak et al., 2013).

All outcome measures were calculated for three successfully recorded trials in each condition of the experiment. Both the mean values and standard deviations over these trials were retained for further analysis. To avoid misinterpretations, “variability” will be used to refer to the magnitude of the standard deviations of the parameters.

### Statistical analysis

A one-way ANOVA was used to compare age, height and weight of typically developing children, children with unilateral and children with bilateral cerebral palsy. A general linear model was used to compare walking speed between subject groups in different walking speed conditions. Herein, subject group was included as a factor (between-subjects) and both arm swing condition (free arm swing or restricted arm swing) and walking speed condition (preferred walking speed or walking “as fast as possible”) were included as repeated measures factors (within-subjects). Moreover, a Mann-Whitney U Test was used to compare children with unilateral cerebral palsy and children with bilateral cerebral palsy for differences regarding the Gross Motor Function Classification Scale-levels and the Modified Ashworth Scale-grades (on the most affected side).

A general linear model was performed to determine the influence of restricting arm swing and increasing walking speed on the outcome parameters, i.e., mean values and the variability of the previously described (1) spatiotemporal parameters; (2) kinematic parameters of trunk movement; (3) margin of stability. The general linear model included subject group as a factor (between-subjects) and arm swing condition (free arm swing or restricted arm swing) and walking speed condition (preferred walking speed or walking “as fast as possible”) as repeated measures factors (within-subjects). To explore group differences regarding the influence of restricting arm swing on gait stability, the arm swing condition ^*^ subject group interactions of the performed general linear models were analyzed. Walking speed condition ^*^ subject group interactions were analyzed to explore group differences regarding the influence of increasing walking speed on gait stability. Similarly, arm swing condition ^*^ walking speed condition ^*^ subject group interactions were analyzed to explore group differences regarding the combined influence of restricting arm swing and increasing walking speed on gait stability. Following the general linear model, Tukey's Honestly Significant Difference-tests were used to perform pairwise comparisons. Moreover, Partial Eta Squared-tests were performed to compute effect sizes for all interactions revealed by the general linear models. Cohen's D-test were performed to compute effect sizes for the pairwise comparisons revealed by the Tukey's Honestly Significant Difference-test.

Statistical analyses were performed using Statistica 8.0 (StatSoft, Inc., USA). The level of significance was set at 0.05 for all tests.

## Results

### Characteristics of typically developing children, children with bilateral cerebral palsy, and children with unilateral cerebral palsy

Group comparisons revealed no differences regarding age and weight (Table 1). However, group differences were found regarding height (*F* = 3.690, *p* = 0.032). Children with unilateral cerebral palsy were significantly smaller than typically developing children (*p* = 0.025). No differences between children with bilateral cerebral palsy and children with unilateral cerebral palsy were found regarding Gross Motor Function Classification System-levels. Children with bilateral cerebral palsy had higher overall median Modified Ashworth Scale-grades on the most affected side compared to children with unilateral cerebral palsy (*Z* = 2.011, *p* = 0.044).

**Table 1:** **Subject characteristics**.

	**Typically developing children**	**Children with bilateral cerebral palsy**	**Children with unilateral cerebral palsy**
*N*	24	15	11
Gender (M/F)	12/12	11/4	8/3
GMFCS (I/II/III)	–	8/6/1^a^	7/4/0
Modified ashworth scale			
Hip flexors		1 (0 − 2); 1 (0 − 2)	0 (0 − 1); 1 (0 − 1+)
Bi-articular hip adductors		1 (0 − 2); 1+ (0 − 2)	0 (0 − 1); 1 (0 − 1+)
Mono-articular hip adductors		1 (0 − 2); 1+ (0 − 2)	0 (0 − 1); 0 (0 − 1.5)
Hamstrings		1+ (0 − 3); 1+ (0 − 3)	1 (0 − 1+); 1+ (1 − 2)
Ankle plantarflexors (measured at 0° knee flexion)		1+ (1 − 2); 2 (0 − 3)	0 (0 − 1+); 2 (0 − 3)
Ankle plantarflexors (measured at 90° knee flexion)		1+ (0 − 2); 1+ (0 − 2)	0 (0 − 1+); 1+ (0 − 2)
Overall median		1+ (0 − 3); 1+ (0 − 3)	0 (0 − 1+); 1 (0 − 3)
Age (y: years, m: months)	9y 5m ± 2y 2m	9y 11m ± 2y 6m	7y 10m ± 3y 0m
Weight (kg)	31.72 ± 8.64	31.54 ± 13.36	23.87 ± 7.57
Height (m)	1.38 ± 0.14	1.34 ± 0.19	1.22 ± 0.15

*Age, weight, and height are presented as follows: mean ± standard deviation. Modified Ashworth Scale values are presented as follows: least affected side median value (least affected side minimum value − least affected side maximum value); most affected side median value (most affected side minimum value − most affected side maximum value)*.

*N, number of subjects; M/F, male/female; GMFCS, Gross Motor Function Classification System*.

a *One subject with GMFCS-level 3 was included because this subject was able to complete the walking trials of the experiment without walking aids*.

### Walking speed in different experimental conditions of typically developing children, children with bilateral cerebral palsy, and children with unilateral cerebral palsy

Statistical analysis revealed a significant walking speed condition ^*^ subject group interaction for walking speed (*F* = 9.901, *p* < 0.001, partial eta-squared = 0.301; Table 2). Walking speed increased more in typically developing children compared to both children with bilateral cerebral palsy and children with unilateral cerebral palsy. This resulted from increased walking speed in typically developing children compared to children with bilateral cerebral palsy at both preferred walking speed (*p* = 0.003, Cohen's *d* = 6.633) and at high walking speed (*p* < 0.001, Cohen's *d* = 9.352). Furthermore, walking speed increased more in typically developing children compared to children with unilateral cerebral palsy. This resulted from increased walking speed in typically developing children compared to children with unilateral cerebral palsy at high walking speed (*p* = 0.004, Cohen's *d* = 4.736), while walking speed was similar at preferred walking speed. This is confirmed by the analysis of the effect sizes: the effect size regarding the increase from the preferred walking speed conditions to the high walking speed conditions is higher for typically developing children (*p* < 0.001, Cohen's *d* = 15.794) compared to both children with bilateral cerebral palsy (*p* < 0.001, Cohen's *d* = 8.042) and children with unilateral cerebral palsy (*p* < 0.001, Cohen's *d* = 8.173).

**Table 2:** **Walking speed in different experimental conditions**.

**Experimental condition**		**Typically developing children (*n* = 24)**	**Children with bilateral cerebral palsy (*n* = 15)**	**Children with unilateral cerebral palsy (*n* = 11)**
“Free arm swing and preferred walking speed”	(m/s)	1.19 ± 0.16	0.94 ± 0.24	1.10 ± 0.13
“Restricted arm swing and preferred walking speed”	(m/s)	1.18 ± 0.16	0.83 ± 0.35	1.01 ± 0.13
“Free arm swing and high walking speed”	(m/s)	1.93 ± 0.26	1.41 ± 0.41	1.67 ± 0.18
“Restricted arm swing and high walking speed”	(m/s)	1.98 ± 0.16	1.35 ± 0.47	1.61 ± 0.16

*Walking speeds are presented as follows: mean ± standard deviation*.

### The influence of restricting arm swing on gait stability in typically developing children, children with bilateral cerebral palsy, and children with unilateral cerebral palsy

#### Spatiotemporal parameters

**Figure 2:**
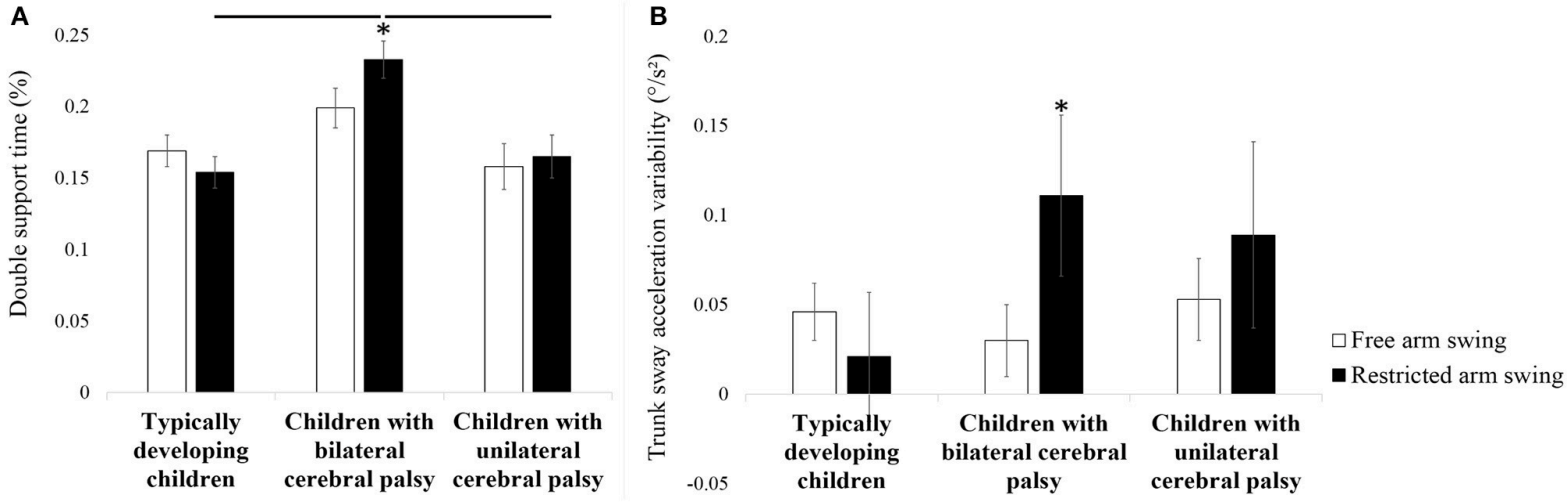
**Influence of restricting arm swing on double support time (A) and trunk sway acceleration variability (B)**. Bars and error bars represent, correspondingly, mean values and standard deviations of the presented outcome parameter for typically developing children, children with bilateral cerebral palsy and children with unilateral cerebral palsy. White (gray) bars represent the values for the free arm swing conditions, black (gray) bars represent the values for the restricted arm swing conditions. An asterisk indicates a significant within subject group difference (*p* < 0.05) in the restricted arm swing conditions compared to the free arm swing conditions (Tukey's pairwise comparisons). A horizontal lines indicates a significant between subject group difference (*p* < 0.05) for the indicated subject groups in the indicated experimental conditions (Tukey's pairwise comparisons).

Statistical analysis revealed a significant arm swing ^*^ subject group interaction for double support time (*F* = 6.164, *p* = 0.004, partial eta-squared = 0.211; Table 3). Double support time increased more in children with bilateral cerebral palsy compared to typically developing children and children with unilateral cerebral palsy. This resulted from a significant increase in double support time in children with bilateral cerebral palsy when arm swing was restricted (*p* = 0.031, Cohen's *d* = 2.517; Figure 2A). Moreover, double support time was higher in children with bilateral cerebral palsy compared to typically developing children (*p* < 0.001, Cohen's *d* = 6.560; Figure 2A) and children with unilateral cerebral palsy walking (*p* = 0.018, Cohen's *d* = 4.845; Figure 2A) when subjects walked with restricted arm swing.

**Table 3:** **Influence of restricting arm swing on different spatiotemporal parameters, kinematic trunk parameters, and margin of stability**.

	**Typically developing children (*****n*** = 24**)**		**Children with bilateral cerebral palsy (*****n*** = 15**)**		**Children with unilateral cerebral palsy (*****n*** = 11**)**		**GLM**
	**Free**	**Restricted**	**Free**	**Restricted**	**Free**	**Restricted**	
Double support time (%)	0.169 ± 0.011	0.154 ± 0.011	0.199 ± 0.014	0.233 ± 0.013	0.158 ± 0.016	0.165 ± 0.015	^*^
Step length (%)	0.448 ± 0.009	0.448 ± 0.011	0.364 ± 0.011	0.354 ± 0.013	0.422 ± 0.013	0.403 ± 0.016	
Step width (%)	0.084 ± 0.008	0.081 ± 0.012	0.102 ± 0.010	0.119 ± 0.015	0.080 ± 0.011	0.090 ± 0.017	
Stride length (%)	0.904 ± 0.018	0.898 ± 0.022	0.714 ± 0.023	0.667 ± 0.028	0.854 ± 0.026	0.834 ± 0.032	
Trunk sway amplitude (°)	5.662 ± 0.905	5.852 ± 0.869	13.717 ± 1.121	13.234 ± 1.076	7.880 ± 1.309	7.320 ± 1.256	
Trunk sway velocity (°/s)	0.354 ± 0.055	0.334 ± 0.045	0.761 ± 0.068	0.700 ± 0.056	0.563 ± 0.080	0.534 ± 0.066	
Trunk sway acceleration (°/s^2^)	0.119 ± 0.031	0.061 ± 0.041	0.130 ± 0.039	0.179 ± 0.051	0.148 ± 0.045	0.208 ± 0.060	^*^
Trunk rotation amplitude (°)	22.192 ± 1.652	22.329 ± 1.915	27.199 ± 2.046	26.226 ± 2.371	26.771 ± 2.389	25.280 ± 2.769	
Trunk rotation velocity (°/s)	1.298 ± 0.115	1.251 ± 0.100	1.649 ± 0.142	1.564 ± 0.124	1.746 ± 0.166	1.708 ± 0.144	
Trunk rotation acceleration (°/s^2^)	0.433 ± 0.125	0.220 ± 0.065	0.575 ± 0.155	0.402 ± 0.080	0.562 ± 0.181	0.609 ± 0.094	
Margin of stability (m)	0.051 ± 0.007	0.053 ± 0.007	0.074 ± 0.008	0.077 ± 0.009	0.064 ± 0.008	0.057 ± 0.009	

Descriptive statistics are presented as mean ± standard deviation for all subjects groups in the free arm swing and the restricted arm swing conditions for all outcome parameters. A general linear model with subject group as a factor and both arm swing condition and walking speed condition as repeated measures factors was performed. An asterisk represents a significant (p < 0.05) subject group ^*^ arm swing interaction effect for the indicated outcome parameter.

*Free, free arm swing conditions; Restricted, restricted arm swing conditions; GLM, general linear model*.

#### Trunk parameters

A significant arm swing ^*^ subject group interaction was found regarding trunk sway acceleration variability (*F* = 4.824, *p* = 0.013, partial eta-squared = 0.173; Table 4). Trunk sway acceleration variability increased more in children with bilateral cerebral palsy compared to typically developing children and children with unilateral cerebral palsy. This resulted from higher trunk sway acceleration variability in children with bilateral cerebral palsy walking with restricted arm swing compared to walking with free arm swing (*p* = 0.045, Cohen's *d* = 2.326; Figure 2B).

**Table 4:** **Influence of restricting arm swing on the variability of different spatiotemporal parameters, kinematic trunk parameters, and margin of stability**.

	**Typically developing children (***n*** = 24**)****		**Children with bilateral cerebral palsy (*****n*** = 15**)**		**Children with unilateral cerebral palsy palsy (*****n*** = 11**)**		**GLM**
	**Free**	**Restricted**	**Free**	**Restricted**	**Free**	**Restricted**	
Double support time (%)	0.035 ± 0.009	0.020 ± 0.007	0.029 ± 0.011	0.045 ± 0.008	0.028 ± 0.012	0.024 ± 0.010	
Step length (%)	0.023 ± 0.002	0.019 ± 0.002	0.028 ± 0.003	0.025 ± 0.003	0.027 ± 0.004	0.026 ± 0.003	
Step width (%)	0.016 ± 0.002	0.015 ± 0.002	0.019 ± 0.003	0.017 ± 0.002	0.017 ± 0.003	0.022 ± 0.003	
Stride length (%)	0.042 ± 0.005	0.035 ± 0.004	0.040 ± 0.006	0.039 ± 0.005	0.038 ± 0.008	0.045 ± 0.006	
Trunk sway amplitude (°)	1.500 ± 0.178	1.535 ± 0.144	2.370 ± 0.220	2.555 ± 0.179	1.705 ± 0.237	1.654 ± 0.209	
Trunk sway velocity (°/s)	0.083 ± 0.013	0.071 ± 0.012	0.123 ± 0.016	0.146 ± 0.015	0.122 ± 0.019	0.106 ± 0.017	
Trunk sway acceleration (°/s^2^)	0.046 ± 0.016	0.021 ± 0.036	0.030 ± 0.020	0.111 ± 0.045	0.053 ± 0.023	0.089 ± 0.052	^*^
Trunk rotation amplitude (°)	4.145 ± 0.534	4.317 ± 0.577	4.429 ± 0.661	4.964 ± 0.714	4.427 ± 0.772	5.860 ± 0.834	
Trunk rotation velocity (°/s)	0.260 ± 0.070	0.251 ± 0.058	0.462 ± 0.087	0.379 ± 0.072	0.441 ± 0.101	0.506 ± 0.084	
Trunk rotation acceleration (°/s^2^)	0.158 ± 0.122	0.071 ± 0.056	0.426 ± 0.152	0.193 ± 0.069	0.276 ± 0.177	0.353 ± 0.081	
Margin of stability (m)	0.019 ± 0.005	0.017 ± 0.004	0.016 ± 0.005	0.018 ± 0.004	0.022 ± 0.005	0.024 ± 0.005	

Descriptive statistics are presented as mean ± standard deviation for all subjects groups in the free arm swing and the restricted arm swing conditions for the variability of all outcome parameters. A general linear model with subject group as a factor and both arm swing condition and walking speed condition as repeated measures factors was performed. An asterisk represents a significant (p < 0.05) subject group ^*^ walking speed interaction effect for the variability of the corresponding outcome parameter.

*Free, free arm swing conditions; Restricted, restricted arm swing conditions; GLM, general linear model*.

#### Margin of stability

No statistically significant group differences were revealed regarding the influence of restricting arm swing on the margin of stability (Tables 3, 4).

### The influence of increasing walking speed on gait stability in typically developing children, children with bilateral cerebral palsy, and children with unilateral cerebral palsy

#### Spatiotemporal parameters

**Figure 3:**
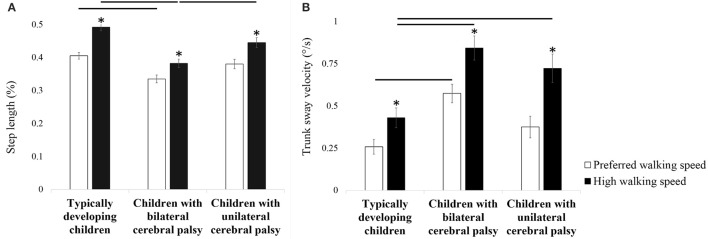
**Influence of increasing walking speed on step length (A) and trunk sway velocity (B)**. Bars and error bars represent, correspondingly, mean values and standard deviations for of the presented outcome parameter for typically developing children, children with bilateral cerebral palsy and children with unilateral cerebral palsy. White bars represent the values for the preferred walking speed conditions, black bars represent the values for the high walking speed conditions. An asterisk indicates a significant within subject group difference (*p* < 0.05) in the high walking speed conditions compared to the preferred walking speed conditions (Tukey's pairwise comparisons). A horizontal line indicates a significant between subject group difference (*p* < 0.05) for the indicated subject groups in the indicated experimental conditions (Tukey's pairwise comparisons).

A significant walking speed condition ^*^ subject group interaction was found for step length (*F* = 5.279, *p* = 0.009, partial eta-squared = 0.187; Table 5). Step length increased more in typically developing children compared to children with bilateral cerebral palsy. This resulted from larger step lengths in typically developing children compared to children with bilateral cerebral palsy at both preferred walking speed (*p* = 0.001, Cohen's *d* = 6.337; Figure 3A) and at high walking speed (*p* < 0.001, Cohen's *d* = 9.135; Figure 3A). Also, step length increased more in children with unilateral cerebral palsy compared to children with bilateral cerebral palsy. This resulted from larger step lengths in children with unilateral cerebral palsy compared to children with bilateral cerebral palsy at high walking speed (*p* = 0.001, Cohen's *d* = 2.120; Figure 3A) while step lengths were similar at preferred walking speed.

**Table 5:** **Influence of increasing walking speed on different spatiotemporal parameters, kinematic trunk parameters, and margin of stability**.

	**Typically developing children (*****n*** = 24**)**		**Children with bilateral cerebral palsy (*****n*** = 15**)**		**Children with unilateral cerebral palsy (*****n*** = 11**)**		**GLM**
	**Preferred**	**High**	**Preferred**	**High**	**Preferred**	**High**	
Double support time (%)	0.197 ± 0.011	0.127 ± 0.010	0.248 ± 0.014	0.185 ± 0.013	0.193 ± 0.016	0.129 ± 0.015	
Step length (%)	0.405 ± 0.010	0.492 ± 0.011	0.335 ± 0.012	0.382 ± 0.013	0.380 ± 0.014	0.445 ± 0.015	^*^
Step width (%)	0.082 ± 0.013	0.083 ± 0.007	0.123 ± 0.016	0.098 ± 0.009	0.089 ± 0.018	0.081 ± 0.011	
Stride length (%)	0.811 ± 0.020	0.991 ± 0.022	0.641 ± 0.025	0.741 ± 0.027	0.780 ± 0.030	0.908 ± 0.032	^*^
Trunk sway amplitude (°)	4.870 ± 0.858	6.644 ± 0.943	12.233 ± 1.062	14.718 ± 1.168	6.223 ± 1.240	8.977 ± 1.364	
Trunk sway velocity (°/s)	0.258 ± 0.044	0.430 ± 0.058	0.574 ± 0.055	0.843 ± 0.072	0.375 ± 0.064	0.723 ± 0.084	^*^
Trunk sway acceleration (°/s^2^)	0.102 ± 0.032	0.078 ± 0.040	0.117 ± 0.039	0.192 ± 0.050	0.127 ± 0.046	0.229 ± 0.058	^*^
Trunk rotation amplitude (°)	15.790 ± 1.507	28.730 ± 1.984	21.889 ± 1.866	31.535 ± 2.457	20.279 ± 2.179	31.772 ± 2.869	
Trunk rotation velocity (°/s)	0.883 ± 0.093	1.666 ± 0.124	1.193 ± 0.115	2.019 ± 0.154	1.249 ± 0.134	2.205 ± 0.180	
Trunk rotation acceleration (°/s^2^)	0.384 ± 0.092	0.268 ± 0.102	0.362 ± 0.113	0.615 ± 0.126	0.466 ± 0.132	0.705 ± 0.147	^*^
Margin of stability (m)	0.046 ± 0.006	0.058 ± 0.008	0.067 ± 0.007	0.084 ± 0.010	0.058 ± 0.007	0.062 ± 0.010	

Descriptive statistics are presented as mean ± standard deviation for all subjects groups in the free arm swing and the restricted arm swing conditions for all outcome parameters. A general linear model with subject group as a factor and both arm swing condition and walking speed condition as repeated measures factors was performed. An asterisk represents a significant (p < 0.05) subject group ^*^ walking speed interaction effect for the indicated outcome parameter.

*Preferred, preferred walking speed conditions; high, high walking speed conditions; GLM, general linear model*.

A significant walking speed condition ^*^ subject group interaction was found for stride length (*F* = 5.950, *p* = 0.005, partial eta-squared = 0.206; Table 5). Stride length increased more in typically developing children compared to children with bilateral cerebral palsy. This resulted from larger stride lengths in typically developing children compared to children with bilateral cerebral palsy at both preferred walking speed (*p* < 0.001, Cohen's *d* = 7.509) and at high walking speed (*p* < 0.001, Cohen's *d* = 10.151). Furthermore, stride length increased more in children with unilateral cerebral palsy compared to children with bilateral cerebral palsy. This resulted from larger stride lengths in children with unilateral cerebral palsy compared to children with bilateral cerebral palsy at both preferred walking speed (*p* = 0.013, Cohen's *d* = 5.034) and at high walking speed (*p* = 0.002, Cohen's *d* = 5.641).

#### Trunk parameters

A significant walking speed condition ^*^ subject group interaction was found for trunk sway velocity (*F* = 5.083, *p* = 0.010, partial eta-squared = 0.181; Table 5). Children with bilateral cerebral palsy increased trunk sway velocity more compared to typically developing children when walking speed was increased. This resulted from higher trunk sway velocity in children with bilateral cerebral palsy compared to typically developing children at both preferred walking speed (*p* = 0.004, Cohen's *d* = 6.345; Figure 3B) and high walking speed (*p* < 0.001, Cohen's *d* = 6.317; Figure 3B). Furthermore, children with unilateral cerebral palsy increased trunk sway velocity more compared to typically developing children when walking speed was increased. This resulted from higher trunk sway velocity at high walking speed in children with unilateral cerebral palsy compared to typically developing children (*p* = 0.025, Cohen's *d* = 4.059; Figure 3B), while trunk sway velocity was similar at preferred walking speed. Moreover, children with unilateral cerebral palsy increased trunk sway velocity more compared to children with bilateral cerebral palsy when walking speed was increased. At high walking speed, trunk sway velocity in children with bilateral cerebral palsy and children with unilateral cerebral palsy was similar. At preferred walking speed, trunk sway velocity was higher in children with bilateral cerebral but similar in children with unilateral cerebral palsy compared to typically developing children. This is confirmed by the analysis of the effect sizes: the effect size regarding the significant increase from the preferred walking speed conditions to the high walking speed conditions is higher for children with unilateral cerebral palsy (*p* < 0.001, Cohen's *d* = 4.660) compared to children with bilateral cerebral palsy (*p* < 0.001, Cohen's *d* = 4.199).

#### Margin of stability

No significant group differences were revealed regarding the influence of increasing walking speed on the margin of stability (Tables 5, 6).

**Table 6:** **Influence of increasing walking speed on the variability of different spatiotemporal parameters, kinematic trunk parameters, and margin of stability**.

	**Typically developing children (*****n*** = 24**)**		**Children with bilateral cerebral palsy (*****n*** = 15**)**		**Children with unilateral cerebral palsy (*****n*** = 11**)**		**GLM**
	**Preferred**	**High**	**Preferred**	**High**	**Preferred**	**High**	
Double support time (%)	0.022 ± 0.002	0.033 ± 0.011	0.028 ± 0.003	0.046 ± 0.013	0.023 ± 0.004	0.029 ± 0.015	
Step length (%)	0.021 ± 0.003	0.021 ± 0.002	0.026 ± 0.003	0.026 ± 0.003	0.031 ± 0.004	0.022 ± 0.003	
Step width (%)	0.015 ± 0.002	0.016 ± 0.002	0.018 ± 0.003	0.018 ± 0.003	0.018 ± 0.003	0.021 ± 0.003	
Stride length (%)	0.036 ± 0.005	0.041 ± 0.005	0.035 ± 0.006	0.043 ± 0.006	0.051 ± 0.007	0.031 ± 0.008	^*^
Trunk sway amplitude (°)	1.166 ± 0.123	1.869 ± 0.188	2.179 ± 0.152	2.747 ± 0.233	1.382 ± 0.177	1.978 ± 0.272	
Trunk sway velocity (°/s)	0.071 ± 0.013	0.083 ± 0.012	0.118 ± 0.016	0.151 ± 0.015	0.091 ± 0.018	0.137 ± 0.018	
Trunk sway acceleration (°/s^2^)	0.045 ± 0.022	0.023 ± 0.030	0.069 ± 0.028	0.071 ± 0.037	0.053 ± 0.032	0.089 ± 0.043	
Trunk rotation amplitude (°)	3.164 ± 0.440	5.298 ± 0.664	3.999 ± 0.545	5.394 ± 0.822	4.259 ± 0.636	6.028 ± 0.960	
Trunk rotation velocity (°/s)	0.225 ± 0.066	0.286 ± 0.054	0.370 ± 0.082	0.470 ± 0.067	0.439 ± 0.096	0.508 ± 0.078	
Trunk rotation acceleration (°/s^2^)	0.155 ± 0.067	0.074 ± 0.109	0.232 ± 0.083	0.387 ± 0.135	0.273 ± 0.097	0.356 ± 0.158	
Margin of stability (m)	0.012 ± 0.004	0.023 ± 0.006	0.015 ± 0.004	0.019 ± 0.006	0.020 ± 0.004	0.025 ± 0.006	

Descriptive statistics are presented as mean ± standard deviation for all subjects groups in the free arm swing and the restricted arm swing conditions for the variability of all outcome parameters. A general linear model with subject group as a factor and both arm swing condition and walking speed condition as repeated measures factors was performed. An asterisk represents a significant (p < 0.05) subject group ^*^ walking speed interaction effect for the variability of the indicated outcome parameter.

*Pref, preferred walking speed conditions; high, high walking speed conditions; GLM, general linear model*.

### The influence of restricting arm swing combined with increasing walking speed on gait stability in typically developing children, children with bilateral cerebral palsy, and children with unilateral cerebral palsy

#### Spatiotemporal parameters

No significant group differences were found regarding the spatiotemporal parameters.

#### Trunk parameters

**Figure 4:**
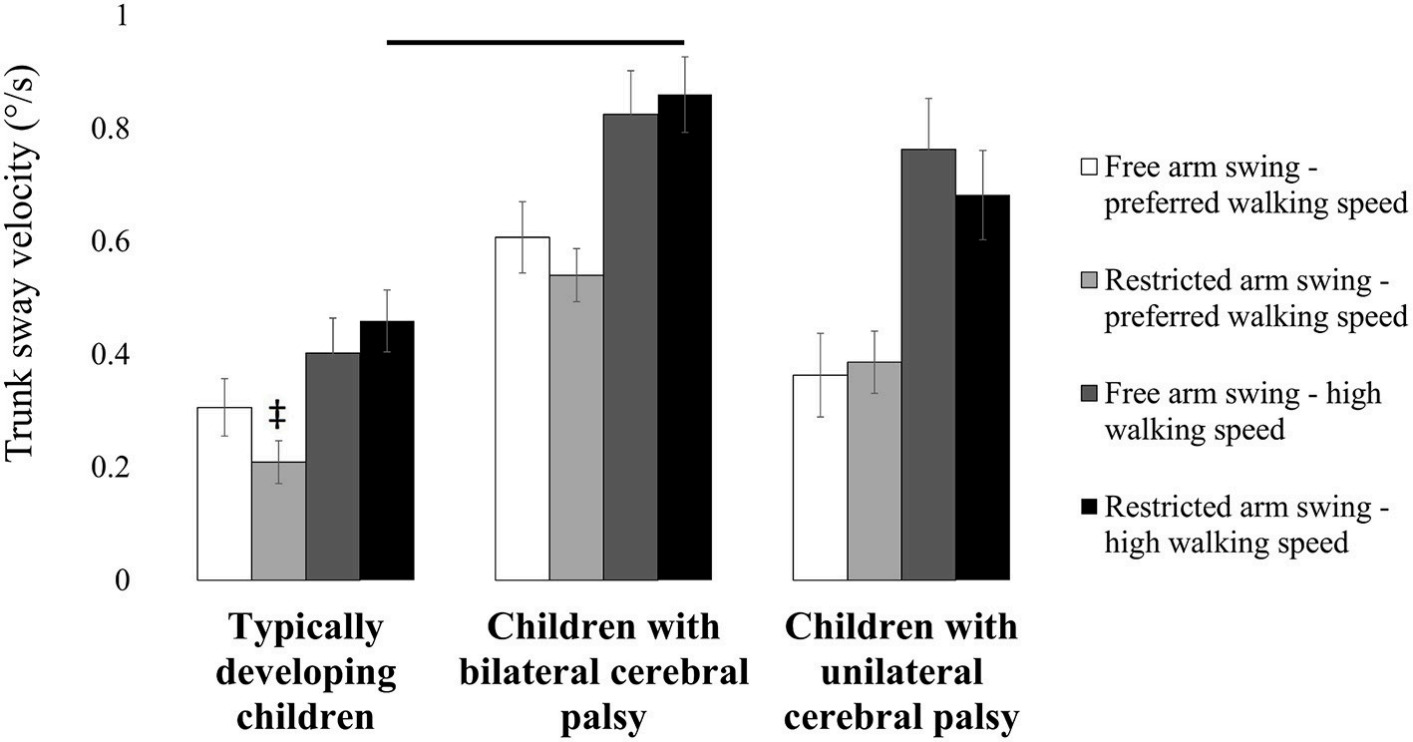
**Influence of restricting arm swing combined with increasing walking speed on trunk sway velocity**. Bars and error bars represent, correspondingly, mean values and standard deviations for trunk sway velocity of typically developing children, children with bilateral cerebral palsy and children with unilateral cerebral palsy. White bars represent the values for “free arm swing and preferred walking speed.” Light gray bars represent the values for “restricted arm swing and preferred walking speed.” Dark gray bars represent the values for “free arm swing and high walking speed.” Black bars represent the values for “restricted arm swing and high walking speed.” A double dagger indicates a significant between subject group difference (*p* < 0.05) in “restricted arm swing and preferred walking speed” compared to “free arm swing and preferred walking speed” (Tukey's pairwise comparisons). A horizontal line indicates a significant between subject group difference (*p* < 0.05) for the indicated subject groups in the indicated experimental conditions (Tukey's pairwise comparisons).

A significant arm swing condition ^*^ walking speed condition ^*^ subject group interaction was observed for trunk sway velocity (*F* = 9.320, *p* < 0.001, partial eta-squared = 0.288; Table 7). Children with bilateral cerebral palsy increased trunk sway velocity more compared to typically developing children from “restricted arm swing and preferred walking speed” to “restricted arm swing and high walking speed.” This resulted from higher trunk sway velocity in “restricted arm swing and high walking speed” in children with bilateral cerebral palsy compared to typically developing children (*p* = 0.033, Cohen's *d* = 6.326; Figure 4), while trunk sway velocity was similar in “restricted arm swing and preferred walking speed.” Besides these different interactions, another interesting difference was observed. Trunk sway velocity was lower in “restricted arm swing and preferred walking speed” compared to “free arm swing and preferred walking speed” for typically developing children (*p* = 0.010, Cohen's *d* = 2.391; Figure 4).

**Table 7:** **Combined influence of restricting arm swing and increasing walking speed on different spatiotemporal parameters, kinematic trunk parameters and margin of stability**.

	**Typically developing children (*****n*** = 24**)**				**Children with bilateral cerebral palsy (*****n*** = 15**)**				**Children with unilateral cerebral palsy (*****n*** = 11**)**				**GLM**
	**Free Pref**.	**Restr. Pref**.	**Free High**	**Restr. High**	**Free Pref**.	**Restr. Pref**.	**Free High**	**Restr. High**	**Free Pref**.	**Restr. Pref**.	**Free High**	**Restr. High**	
Double support time (%)	0.207 ± 0.010	0.186 ± 0.013	0.131 ± 0.014	0.123 ± 0.012	0.231 ± 0.013	0.264 ± 0.016	0.167 ± 0.017	0.202 ± 0.014	0.190 ± 0.015	0.195 ± 0.019	0.125 ± 0.020	0.134 ± 0.017	
Step length (%)	0.404 ± 0.009	0.406 ± 0.012	0.493 ± 0.011	0.491 ± 0.011	0.341 ± 0.011	0.328 ± 0.015	0.386 ± 0.013	0.379 ± 0.014	0.388 ± 0.013	0.372 ± 0.018	0.455 ± 0.015	0.434 ± 0.014	
Step width (%)	0.083 ± 0.006	0.081 ± 0.013	0.084 ± 0.005	0.081 ± 0.006	0.107 ± 0.008	0.138 ± 0.017	0.098 ± 0.007	0.099 ± 0.007	0.088 ± 0.009	0.090 ± 0.020	0.073 ± 0.008	0.089 ± 0.008	
Stride length (%)	0.810 ± 0.018	0.812 ± 0.025	0.999 ± 0.022	0.984 ± 0.023	0.678 ± 0.022	0.604 ± 0.031	0.750 ± 0.027	0.731 ± 0.028	0.795 ± 0.026	0.765 ± 0.036	0.913 ± 0.032	0.903 ± 0.033	^*^
Trunk sway amplitude (°)	5.108 ± 0.939	4.632 ± 0.811	6.216 ± 0.957	7.072 ± 0.999	12.818 ± 1.162	11.649 ± 1.005	14.615 ± 1.186	14.820 ± 1.237	6.366 ± 1.357	6.080 ± 1.173	9.394 ± 1.384	8.560 ± 1.445	
Trunk sway velocity (°/s)	0.306 ± 0.053	0.209 ± 0.039	0.402 ± 0.063	0.459 ± 0.056	0.607 ± 0.066	0.540 ± 0.048	0.825 ± 0.078	0.860 ± 0.070	0.363 ± 0.077	0.386 ± 0.056	0.763 ± 0.091	0.682 ± 0.082	^*^
Trunk sway acceleration (°/s^2^)	0.171 ± 0.030	0.033 ± 0.025	0.067 ± 0.024	0.089 ± 0.036	0.092 ± 0.037	0.143 ± 0.031	0.168 ± 0.030	0.216 ± 0.044	0.079 ± 0.043	0.175 ± 0.037	0.218 ± 0.034	0.240 ± 0.052	^*^
Trunk rotation amplitude (°)	17.464 ± 1.167	14.116 ± 1.137	26.919 ± 1.451	30.541 ± 1.949	23.761 ± 1.445	20.017 ± 1.408	30.637 ± 1.797	32.434 ± 2.413	21.968 ± 1.687	18.591 ± 1.645	31.574 ± 2.098	31.970 ± 2.818	
Trunk rotation velocity (°/s)	1.134 ± 0.124	0.631 ± 0.090	1.461 ± 0.133	1.871 ± 0.150	1.237 ± 0.153	1.150 ± 0.112	2.061 ± 0.165	1.978 ± 0.186	1.244 ± 0.179	1.255 ± 0.131	2.249 ± 0.193	2.161 ± 0.217	^*^
Trunk rotation acceleration (°/s^2^)	0.658 ± 0.131	0.111 ± 0.082	0.207 ± 0.155	0.329 ± 0.069	0.371 ± 0.162	0.353 ± 0.102	0.779 ± 0.192	0.451 ± 0.086	0.373 ± 0.189	0.558 ± 0.119	0.750 ± 0.224	0.660 ± 0.100	^*^
Margin of stability (m)	0.045 ± 0.004	0.047 ± 0.005	0.057 ± 0.006	0.058 ± 0.007	0.064 ± 0.005	0.069 ± 0.006	0.083 ± 0.008	0.085 ± 0.008	0.062 ± 0.005	0.055 ± 0.006	0.066 ± 0.008	0.059 ± 0.008	

Descriptive statistics are presented as mean ± standard deviation for all subjects groups in all experimental conditions for all outcome parameters. A general linear model with subject group as a factor and both arm swing condition and walking speed condition as repeated measures factors was performed. An asterisk represents a significant (p < 0.05) subject group ^*^ arm swing condition ^*^ walking speed interaction effect for the variability of the indicated outcome parameter.

*Free, free arm swing condition; Restr, restricted arm swing condition; Pref, preferred walking speed condition; High, high walking speed condition; GLM, general linear model*.

**Figure 5:**
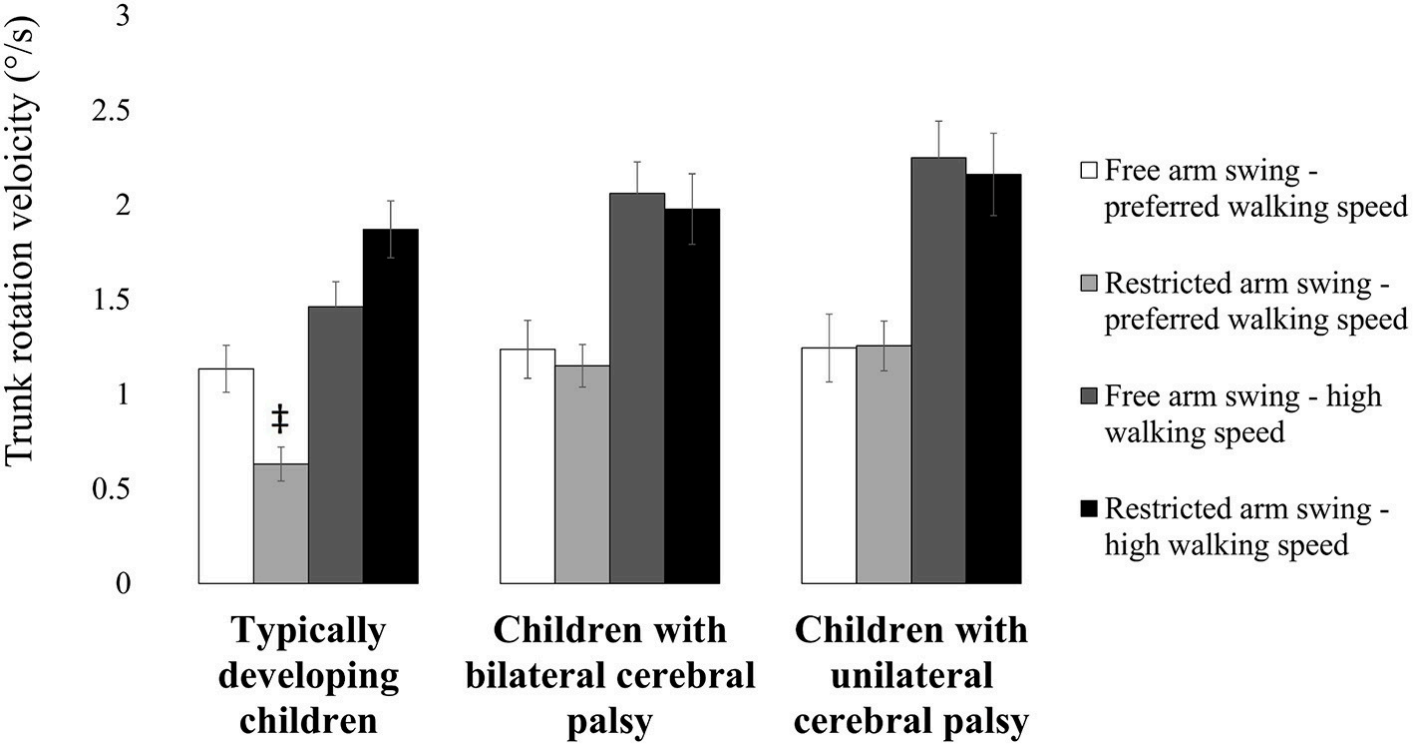
**Influence of restricting arm swing combined with increasing walking speed on trunk rotation velocity**. Bars and error bars represent, correspondingly, mean values and standard deviations for trunk rotation velocity of typically developing children, children with bilateral cerebral palsy and children with unilateral cerebral palsy. White bars represent the values for “free arm swing and preferred walking speed.” Light gray bars represent the values for “restricted arm swing and preferred walking speed.” Dark gray bars represent the values for “free arm swing and high walking speed.” Black bars represent the values for “restricted arm swing and high walking speed.” A double dagger indicates a significant between subject group difference (*p* < 0.05) in “restricted arm swing and preferred walking speed” compared to “free arm swing and preferred walking speed” (Tukey's pairwise comparisons).

Statistical analysis also revealed a significant arm swing condition ^*^ walking speed condition ^*^ subject group interaction for trunk rotation velocity (*F* = 6.976, *p* = 0.002, partial eta-squared = 0.233; Table 7). Typically developing children increased trunk rotation velocity more from “restricted arm swing and preferred walking speed” to “restricted arm swing and high walking speed” compared to children with bilateral cerebral palsy and unilateral cerebral palsy. This resulted from lower trunk rotation velocity in “restricted arm swing and preferred walking speed” compared to “free arm swing and preferred walking speed” in typically developing children (*p* = 0.018, Cohen's *d* = 4.634; Figure 5). Trunk rotation velocity was similar in “free arm swing and preferred walking speed” and in “restricted arm swing and high walking speed” for all three subject groups.

#### Margin of stability

Analysis of the arm swing ^*^ walking speed ^*^ group interactions of the margin of stability revealed no significant differences (Table 7). Moreover, no significant differences were found for the arm swing ^*^ walking speed ^*^ group interactions of the variability of the margins of stability (Table 8).

**Table 8:** **Combined influence of restricting arm swing and increasing walking speed on the variability of different spatiotemporal parameters, kinematic trunk parameters, and margin of stability**.

	**Typically developing children (*****n*** = 24**)**				**Children with bilateral cerebral palsy (*****n*** = 15**)**				**Children with unilateral cerebral palsy (*****n*** = 11**)**				**GLM**
	**Free Pref**.	**Restr. Pref**.	**Free High**	**Restr. High**	**Free Pref**.	**Restr. Pref**.	**Free High**	**Restr. High**	**Free Pref**.	**Restr. Pref**.	**Free High**	**Restr. High**	
Double support time (%)	0.022 ± 0.002	0.022 ± 0.003	0.049 ± 0.017	0.018 ± 0.014	0.026 ± 0.003	0.030 ± 0.004	0.031 ± 0.021	0.060 ± 0.017	0.022 ± 0.004	0.023 ± 0.005	0.033 ± 0.024	0.024 ± 0.020	
Step length (%)	0.022 ± 0.004	0.020 ± 0.003	0.024 ± 0.003	0.019 ± 0.003	0.028 ± 0.005	0.025 ± 0.004	0.028 ± 0.004	0.024 ± 0.003	0.035 ± 0.005	0.027 ± 0.005	0.020 ± 0.005	0.025 ± 0.004	
Step width (%)	0.014 ± 0.002	0.016 ± 0.002	0.018 ± 0.002	0.014 ± 0.002	0.019 ± 0.002	0.017 ± 0.003	0.019 ± 0.003	0.016 ± 0.002	0.012 ± 0.003	0.024 ± 0.003	0.022 ± 0.003	0.020 ± 0.003	
Stride length (%)	0.036 ± 0.005	0.036 ± 0.004	0.048 ± 0.006	0.034 ± 0.005	0.036 ± 0.006	0.035 ± 0.006	0.043 ± 0.007	0.043 ± 0.006	0.049 ± 0.007	0.054 ± 0.006	0.026 ± 0.008	0.037 ± 0.007	
Trunk sway amplitude (°)	1.123 ± 0.167	1.209 ± 0.150	1.877 ± 0.251	1.862 ± 0.240	2.015 ± 0.207	2.342 ± 0.186	2.726 ± 0.310	2.768 ± 0.297	1.428 ± 0.242	1.336 ± 0.217	1.982 ± 0.362	1.973 ± 0.346	
Trunk sway velocity (°/s)	0.080 ± 0.015	0.062 ± 0.016	0.086 ± 0.017	0.080 ± 0.015	0.101 ± 0.018	0.136 ± 0.020	0.146 ± 0.021	0.156 ± 0.019	0.092 ± 0.021	0.091 ± 0.024	0.151 ± 0.024	0.122 ± 0.022	
Trunk sway acceleration (°/s^2^)	0.070 ± 0.014	0.019 ± 0.026	0.022 ± 0.014	0.024 ± 0.032	0.022 ± 0.018	0.116 ± 0.032	0.037 ± 0.017	0.105 ± 0.040	0.022 ± 0.021	0.084 ± 0.037	0.083 ± 0.020	0.095 ± 0.046	
Trunk rotation amplitude (°)	3.178 ± 0.338	3.150 ± 0.454	5.112 ± 0.602	5.484 ± 0.610	3.524 ± 0.418	4.473 ± 0.562	5.334 ± 0.746	5.455 ± 0.755	3.599 ± 0.489	4.919 ± 0.656	5.254 ± 0.871	6.801 ± 0.882	
Trunk rotation velocity (°/s)	0.278 ± 0.082	0.172 ± 0.096	0.241 ± 0.089	0.330 ± 0.046	0.349 ± 0.102	0.392 ± 0.119	0.576 ± 0.110	0.365 ± 0.057	0.385 ± 0.119	0.493 ± 0.139	0.496 ± 0.129	0.519 ± 0.067	
Trunk rotation acceleration (°/s^2^)	0.257 ± 0.082	0.052 ± 0.081	0.058 ± 0.183	0.089 ± 0.054	0.247 ± 0.102	0.218 ± 0.100	0.605 ± 0.226	0.168 ± 0.067	0.143 ± 0.119	0.402 ± 0.117	0.409 ± 0.264	0.303 ± 0.078	
Margin of stability (m)	0.012 ± 0.003	0.013 ± 0.004	0.026 ± 0.006	0.021 ± 0.004	0.015 ± 0.003	0.016 ± 0.004	0.018 ± 0.006	0.020 ± 0.004	0.020 ± 0.003	0.021 ± 0.005	0.023 ± 0.007	0.026 ± 0.005	

Descriptive statistics are presented as mean ± standard deviation for all subjects groups in all experimental conditions for all outcome parameters. A general linear model with subject group as a factor and both arm swing condition and walking speed condition as repeated measures factors was performed. No significant (p < 0.05) subject group ^*^ arm swing condition ^*^ walking speed interaction effects were found for the variability of the presented outcome parameters.

*Free, free arm swing condition; Restr, restricted arm swing condition; Pref, preferred walking speed condition; High, high walking speed condition; GLM, general linear model*.

## Discussion

In the current study, the influence of restricting arm swing and increasing walking speed in typically developing children and children with both unilateral and bilateral cerebral palsy was compared to gain insight on the stabilizing role of arm swing during walking. First, it was hypothesized that gait stability would decrease more in children with cerebral palsy compared to typically developing children when arm swing is restricted. It was also expected that gait stability would decrease more in children with bilateral cerebral palsy compared to children with unilateral cerebral palsy when arm swing is restricted. Second, it was hypothesized that gait stability would decrease more in children with cerebral palsy compared to typically developing children when walking speed is increased. It was also expected that gait stability would decrease more in children with bilateral cerebral palsy compared to children with unilateral cerebral palsy when walking speed is increased. Finally, it was hypothesized that gait stability would decrease more in children with cerebral palsy compared to typically developing children when both arm swing is restricted and walking speed is increased. It was also expected that gait stability would decrease more in children with bilateral cerebral palsy compared to children with unilateral cerebral palsy when both arm swing is restricted and walking speed is increased. Additionally, it was expected that the influence of restricting arm swing combined with increasing walking speed would be larger compared to the isolated influence of these tasks.

### The influence of restricting arm swing on gait stability in typically developing children, children with bilateral cerebral palsy, and children with unilateral cerebral palsy

Previous research suggested that altered arm postures in children with cerebral palsy were related to gait instability (Meyns et al., 2012a). The current results partly support these observational findings.

Double support time increased more in children with bilateral cerebral palsy compared to typically developing children and children with unilateral cerebral palsy when arm swing was restricted. Children with bilateral cerebral palsy may have tried to enhance stability of walking by minimizing the impact of an instable single support phase (Kim and Son, 2014). Therefore, the larger increase in double support time in the children with cerebral palsy (when they are not allowed to freely swing their arms) is considered as an indication for the stabilizing role of arm swing in children with bilateral cerebral palsy.

The larger increase in trunk sway acceleration variability in children with bilateral cerebral palsy also suggests an increase in gait instability when arm swing is restricted. Measures of kinematic variability have been extensively used to quantify gait stability (Bruijn et al., 2013a). For instance, measures of trunk acceleration variability reliably estimated the risk of falls in elderly (Doi et al., 2013). Higher trunk acceleration variability could decrease gait stability through the disturbance of optic flow and vestibular signals (Holt et al., 1999; Iosa et al., 2014). Therefore, the larger increase in trunk acceleration variability in children with bilateral cerebral palsy when arm swing was restricted is also considered as an indication for the stabilizing role of arm swing in children with bilateral cerebral palsy.

Increased trunk sway acceleration variability in children with bilateral cerebral palsy could also be explained by trunk control deficits in children with cerebral palsy (Heyrman et al., 2011, 2014; Attias et al., 2015). Trunk control deficits have been shown to be strongly correlated to the level of impairment (Attias et al., 2015) and to increased range of motion of trunk movements in children with cerebral palsy (Heyrman et al., 2013, 2014). However, the children with cerebral palsy included in the study were only mildly impaired. Therefore, trunk control deficits in the current population can be expected to be mild. Furthermore, no differences were observed regarding trunk sway and trunk rotational range of motion. Although the adopted computational methods may differ, the values reported in literature (Heyrman et al., 2013; Attias et al., 2015) are similar to the values reported in the current study. Therefore, it is assumed that, in the mildly impaired subjects included in the current study, trunk control deficits did not primarily cause the group differences in trunk sway acceleration variability when arm swing was restricted.

Next to possible trunk control deficits, altered angular momentum in children with cerebral palsy could also partly explain the observed group differences regarding trunk kinematics. Previous research indicated that arm swing movements compensated for the angular momentum (around a vertical axis) disruptions during walking by the involved leg in children with unilateral cerebral palsy (Bruijn et al., 2011). Possibly, restricting arm swing forces the trunk to take over the role of the arms in compensating angular momentum disruptions. However, other research found that angular momentum around the vertical axis was similar for children with bilateral cerebral palsy and typically developing children during walking (Russell et al., 2011). Therefore, it is assumed that, when arm swing was restricted, trunk compensation for angular momentum disruptions around the vertical axis did not primarily cause the group differences regarding increased trunk sway acceleration variability.

It is remarkable that no differences were detected regarding the influence of restricting arm swing on gait stability for children with unilateral cerebral palsy compared to typically developing children. It seems that restricting arm swing did not sufficiently challenge children with unilateral cerebral palsy to increase gait instability more compared to typically developing children. Therefore, it is assumed that the role of arm swing in gait stability is smaller in children with unilateral cerebral palsy compared to children with bilateral cerebral palsy.

Furthermore, no changes in margins of stability and step width were found when arm swing was restricted (nor when walking speed was increased; see below). This possibly suggests that gait stability is very mildly affected. On the other hand, it is possible that restricting arm swing affects gait stability in a specific direction. Previous research indicated that children with unilateral cerebral palsy show gait instability in both the medio-lateral and the antero-posterior direction (Bruijn et al., 2013b). Since restricting arm swing did not affect the margins of stability nor step width, it seems reasonable to suggest that restricting arm swing does not specifically affect medio-lateral gait stability.

In conclusion, children with bilateral cerebral palsy showed larger increases in double support time and trunk sway acceleration variability compared to typically developing children and children with unilateral cerebral palsy. As hypothesized, these findings suggest that arm swing has a stabilizing role during gait in children with bilateral cerebral palsy. Trunk control deficits and trunk compensations for disrupted angular momentum are also suggested to influence gait instability in children with bilateral cerebral palsy (although to a smaller degree). Furthermore, the hypothesis that restricting arm swing would decrease gait stability more in children with bilateral cerebral palsy compared to children with unilateral cerebral palsy seems to be confirmed.

### The influence of increasing walking speed on gait stability in typically developing children, children with bilateral cerebral palsy, and children with unilateral cerebral palsy

Since we aimed to evaluate the combined effect of increasing walking speed and restricting arm swing, the isolated influence of walking speed on the measures of stability is described first.

Typically developing children increased step length and stride length more compared to children with bilateral cerebral palsy when walking speed was increased. Children with cerebral palsy face muscle shortening, muscle contractures and/or spasticity. Since spasticity is dependent of muscle lengthening velocity, this could influence step length more when increasing walking speed. Children with unilateral cerebral palsy increased step length more compared to children with bilateral cerebral palsy when walking speed was increased. This difference is likely to be explained by the lower spasticity values in children with unilateral cerebral palsy and bilateral involvement in children with bilateral cerebral palsy (in contrast to unilateral involvement in children with unilateral cerebral palsy).

The reported group differences regarding the increase in trunk sway velocity when walking speed was increased could also be explained by compensations for differences regarding the increase in step length and stride length. However, step (stride) length increased more in children with unilateral cerebral palsy compared to children with bilateral cerebral palsy. If step (stride) length primarily caused the reported group differences regarding trunk sway velocity, one would expect a smaller increase in trunk sway velocity in children with bilateral cerebral palsy compared to children with unilateral cerebral palsy. Clearly, this is not supported by the results. Moreover, previous research indicated that altered trunk movements in children with cerebral palsy are not likely to be compensations due to lower limb impairments (Heyrman et al., 2014). Therefore, it is assumed that trunk compensation for muscle spasticity in the legs did not primarily cause the group differences regarding increased trunk sway velocity when walking speed was increased.

Furthermore, increased trunk sway acceleration variability in children with bilateral cerebral palsy could also be explained by trunk control deficits in children with cerebral palsy (Heyrman et al., 2011, 2014; Attias et al., 2015). However, it is assumed that, in the mildly impaired subjects included in the current study, trunk control deficits did not primarily cause the group differences in trunk sway acceleration variability when arm swing was restricted. A profound elaboration can be found in the previous section.

Additionally, the reported group differences regarding the increase in trunk sway velocity when walking speed was increased could be explained by differences regarding the angular momentum around the vertical axis. Both children with bilateral cerebral palsy and children with unilateral cerebral palsy increased trunk sway velocity more when walking speed was increased. Previous research already indicated that the angular momentum around the vertical of the unaffected arm and leg in children with unilateral cerebral palsy were higher compared to typically developing children (Bruijn et al., 2011). Furthermore, upper body angular momentum around the vertical axis was higher in children with bilateral cerebral palsy compared to typically developing children (Russell et al., 2011). Moreover, previous research indicates that the angular momentum of body segments around a vertical axis increases with walking speed (Bruijn et al., 2008). As such, the larger increase in trunk sway velocity could be explained by the larger increase in walking speed in children with unilateral cerebral palsy compared to children with bilateral cerebral palsy. Therefore, the group differences regarding the increase in trunk sway velocity when walking speed was increased are considered as an indication for trunk compensational movements for angular momentum disruptions.

In conclusion, children with cerebral palsy showed larger increases regarding trunk sway velocity when walking speed was increased compared to typically developing children. Furthermore, children with unilateral cerebral palsy increased trunk sway velocity more compared to children with bilateral cerebral palsy when walking speed was increased. It is proposed that the group differences regarding the increase in trunk sway velocity when walking speed was increased may be considered as an indication for trunk compensational movements for angular momentum disruptions. Trunk control deficits, trunk compensations for muscle spasticity and impaired step length are also suggested to influence trunk sway velocity in children with bilateral cerebral palsy (although to a smaller degree). In contrast to the research hypothesis, gait stability did not decrease more in children with cerebral palsy compared to typically developing children when walking speed was increased.

### The influence of restricting arm swing combined with increasing walking speed on gait stability in typically developing children, children with bilateral cerebral palsy, and children with unilateral cerebral palsy

An important group difference regarding the combined influence of restricting arm swing and increasing walking speed was found (similar to the isolated influence of increasing walking speed). A stronger increase in trunk sway velocity has been observed in children with bilateral cerebral palsy compared to typically developing children when subjects walking with restricted arm swing were asked to increase walking speed. This possibly suggests that increasing walking speed combined with restricting arm swing decreases gait stability more in children with bilateral cerebral palsy compared to typically developing children. However, these findings should certainly be interpreted with care because other factors (trunk control deficits, altered angular momentum and compensations for lower limb impairments) may interfere with the combined influence of arm swing and walking speed on gait stability (as mentioned above).

Furthermore, typically developing children showed a specific reaction when arm swing was restricted at preferred walking speed (and not at increased walking speed). Both trunk sway and trunk rotation decreased compared to walking with the arms free at the preferred walking speed. This “en bloc” strategy was not found in either group of children with cerebral palsy. Therefore, it is assumed that the trunk is required to move in children with cerebral palsy when arm swing is restricted.

In conclusion, children with bilateral cerebral palsy increased trunk sway velocity more compared to typically developing children when subjects walking with restricted arm swing were asked to increase walking speed. Overall, evidence is insufficient to conclude that restricting arm swing combined with increasing walking speed induced larger group differences regarding gait stability compared to their isolated effects. Thereby, the experimental data could not confirm the postulated research hypotheses regarding the influence of both restricted arm swing and increased walking speed on gait stability. However, the results showed that children with cerebral palsy adopted different responses to arm swing restriction compared to typically developing children regarding trunk kinematics.

### Limitations to the current research

When interpreting the results of the current study, certain methodological issues should be taken into account. It is possible that the study sample consisting of 24 typically developing children and 26 children with cerebral palsy was too small and/or heterogeneous. This could have caused some marginally non-significant changes that were reported. Additionally, more children with bilateral cerebral palsy had a GMFCS level II than children with unilateral cerebral palsy. These differences certainly need to be taken into account when comparing these two groups. Most of the subjects included in the group of children with cerebral palsy had a GMFCS level I. This mildly involved population could have caused an underestimation of the actual differences between typically developing children and children with cerebral palsy.

Second, Bruijn et al. (2013a) reported a vast amount of possible parameters to assess stability of gait. Since gait stability is a multifactorial concept, not all parameters measure the same part of this concept and different parameters of gait stability possibly reflect different and contrasting viewpoints. For instance, previous research did not find a correlation between a measure of local dynamic stability and step length variability (Kurz et al., 2012). Therefore, further research to validate and to frame these measures in the global concept of gait stability needs to be conducted (Bruijn et al., 2013a). Research for measures of gait stability with proven validity and reliability in children with cerebral palsy specifically and across different population groups is still needed.

Finally, trunk movements were not described using joint angles. However, elevation angles were used (i.e., the angle between segments projected in one plane). As such, a simplified kinematic method was used. In previous research, this approach was found to be adequate to detect meaningful changes in the kinematics during walking in this population, in agreement with literature using joint angles (Meyns et al., 2011, 2012a,b).

## Conclusion

In conclusion, restricting arm swing influenced gait stability more in children with bilateral cerebral palsy compared to both typically developing children and children with unilateral cerebral palsy. As such, the current study is the first to support experimentally that arm swing compensates (at least partly) for affected stability in children with bilateral cerebral palsy. Results were less clear for children with unilateral cerebral palsy. In contrast to the research hypotheses, increasing walking speed did not affect gait stability more in children with cerebral palsy compared to typically developing children (nor in children with bilateral cerebral palsy compared to children with unilateral cerebral palsy). However, the current results indicate that increasing walking speed increased trunk compensations for altered angular momentum around a vertical axis more in children with cerebral palsy compared to typically developing children. The effects were larger in children with unilateral cerebral palsy compared to children with bilateral cerebral palsy because more trunk movements were already observed in children with bilateral cerebral palsy at preferred walking speed. In contrast to the research hypotheses, restricting arm swing combined with increasing walking speed did not induce larger group differences regarding gait stability compared to their isolated effects. Overall, it is proposed that facilitating arm swing during gait rehabilitation can improve gait stability and decrease trunk movements in children with cerebral palsy. The current results partly support the suggestion that facilitating arm swing in specific situations possibly enhances safety and reduce the risk of falling in children with cerebral palsy. Other authors already suggested a similar approach for other pathologies (e.g., stroke and spinal cord injury) in previous research (Stephenson et al., 2010; Tester et al., 2011). Moreover, previous research of our research group in a cerebral palsy population supports this conclusion (Meyns et al., 2011).

## Author contributions

PM and KD conceived and designed the experiment. PM performed the experiments. PM and TD together analyzed the data. Furthermore, TD, PM, and KD wrote the paper. The writing process and the data analysis were supervised by PM and KD.

## Funding

This project was supported by grants from the “bijzonder onderzoeksfonds” KU Leuven (OT/08/034 & PDMK/12/180) and from the Research Foundation Flanders (FWO grant G.0901.11; “Krediet aan Navorser” grant 1503915N). PM is supported by the European Commission (Horizon 2020) as a Marie Skłodowska-Curie fellow (proposal 660458). The funding agencies had no role in the present study.

### Conflict of interest statement

The authors declare that the research was conducted in the absence of any commercial or financial relationships that could be construed as a potential conflict of interest.
